# Effectiveness of Alcohol, Smoking, and Substance Involvement Screening Test-Linked Brief Intervention on Harmful and Hazardous Alcohol Use in Nigerian Semirural Communities: A Non-Randomized Intervention Study

**DOI:** 10.3389/fpsyt.2017.00050

**Published:** 2017-04-10

**Authors:** Victor Lasebikan, Bolanle Adeyemi Ola, Olatunde O. Ayinde

**Affiliations:** ^1^Department of Psychiatry, College of Medicine, University of Ibadan, Ibadan, Nigeria; ^2^Department of Psychiatry, University College Hospital, Ibadan, Nigeria; ^3^Department of Behavioural Sciences, College of Medicine, Lagos State University, Lagos, Nigeria

**Keywords:** alcohol, rural communities, effectiveness, health risks, screening, brief intervention

## Abstract

**Objective:**

To determine the prevalence of alcohol consumption and the effectiveness of the alcohol, smoking, and substance involvement screening test (ASSIST)-linked brief intervention on hazardous and harmful alcohol use in semirural settings in Nigeria.

**Methods:**

In this single arm non-randomized intervention study delivered by community health extension workers (CHEW), participants (*N* = 1,203), 15 years and older, recruited between October 2010 and April 2011 were assessed for prevalence of alcohol consumption and the associated level of risk. Scores of 0–10 were classified as lower risk scores, 11–26 as moderate risk, and 27+ as high risk. This was followed by a brief intervention. Prevalence of alcohol consumption and level of risk was assessed at 3 and 6 months postbrief intervention. Main outcome measure was the change in ASSIST scores at 3 and 6 months postintervention.

**Results:**

There was a statistically significant difference in the prevalence of alcohol use at baseline compared with that at 6 months, χ^2^(2) = 4.2, *p* = 0.01. Among all respondents, a repeated measures ANOVA with a Greenhouse–Geisser correction showed that mean ASSIST score significantly reduced between time points [*F*(1.541, 34.092) = 53.241, *p* < 0.001]. *Post hoc* tests using the Bonferroni correction revealed that this difference was due to a significant reduction in the mean ASSIST scores at 3 months vs. baseline, *p* = 0.001, but not at 3 vs. 6 months, *p* = 0.09.

**Conclusion:**

There is a potential for CHEW-administered ASSIST-linked screening, brief intervention, and referral to treatment for unhealthy alcohol use in Nigerian semirural communities. This is feasible considering serious dearth of addiction specialists in the country.

## What is Known

- Problem alcohol use is a huge public health issue worldwide and among Nigerian adults.
- Screening, brief intervention, and referral for treatment (SBIRT) is an evidence-based model for reduction in alcohol consumption in primary care health settings in the Western world.

## What the Study Adds

- The efficacy of community health extension worker delivered SBIRT ASSIST-linked intervention on problem alcohol use in a sub-Saharan rural community setting has been demonstrated.

## Introduction

Alcohol increases the odds of negative health outcomes worldwide (1–3) and ranks among the top causes of early deaths (4). It is a factor in a large proportion of injuries (5, 6) and is also associated with psychiatric morbidities (7, 8), alcohol use disorders (9, 10), and medical comorbidities (11).

Screening, brief intervention, and referral to treatment (SBIRT) is an evidence-based public health approach aimed at early intervention and treatment services for individuals with substance use disorders including those at risk (12). The current model of SBIRT is based on the Institute of Medicine report, which recommended: “the development of integrated service systems that link community-based screening and brief intervention to assessment and referral activities” (13). The primary objective is to reduce the prevalence and adverse consequences of substance misuse and substance use disorders, thereby improving community health. SBIRT was developed originally for alcohol screening and brief intervention in primary health care settings (12). Several meta-analyses abound to support the efficacy of SBIRT in leading to a significant reduction of alcohol consumption in primary care population in Western countries and these efforts impacted positively on health care delivery systems for per-sons with substance abuse, those at risk, and substance abuse policy (14–16).

There is a dearth of data on the efficacy of SBIRT in rural community settings in sub-Saharan Africa that could usefully translate into substance abuse policy with impact on at-risk populations as well as those who substance use problems. However, few studies have examined the efficacy of SBIRT in South Africa. These demonstration projects were part of a WHO strategy to address alcohol problems in developing countries and were conducted in health care settings that included hospital outpatients (17, 18) and rural settings (19). These studies demonstrated that nurses and community health workers were strategic implementation agents in health care settings (17). There are good evidence and compelling logic to support the allocation of tasks in health-system delivery to the least costly health worker capable of doing that task reliably (20). This task-shifting approach had been found to be applicable in low-resource countries, where the use of health workers with a shorter duration of training performed at least as well and sometimes substantially better than those with a longer duration of training (21).

In Nigeria, however, while there are indications that there has been a rapid increase in alcohol availability and consumption across all age groups (22), data on alcohol consumption in the rural communities is sparse, as well as effective evidence-based intervention, despite evidences that drinking rates are higher in the rural areas (19). Unfortunately, the majority of Nigerians live in semirural or rural settings, and manpower and resources are poor. Against the backdrop of service gaps in underserved rural areas, the concept of task shifting could be applicable. A recent situational analysis suggests that Nigeria has made considerable gains with task shifting in some medical specialties, where dedicated low-cost health workers at the community and clinic levels supplement integrated care. Dedicated low-cost health care workers such as community health extension workers (CHEW) are available and more in contact with people at the grass roots especially with regards to immunization programmes in Nigeria. They have been involved in task-shifting models to address gaps in reproductive health care in primary care settings in Nigeria (23) as well as for depression in primary health care settings (24). However, this model has not been examined with regards to substance use and particularly in rural settings. Task-shifting screening and brief intervention for hazardous drinking to CHEWs may be a viable, cost-effective option in addressing the rising prevalence of problem drinking in Nigeria. This study aimed to conduct a single arm trial to assess the effectiveness of CHEW-delivered SBIRT for alcohol use among people in semirural community settings. We hypothesized that CHEW-administered alcohol, smoking, and substance involvement screening test (ASSIST)-linked SBIRT would significantly reduce the prevalence of alcohol use in Nigerian semirural settings.

## Materials and Methods

We report herein, the findings of the outcome of the brief intervention delivered to rural dwellers in Ibadan, Nigeria. The methodology and the results of the baseline assessments have been previously reported (25).

### Study Area

The study site was in Ibadan, Oyo State. Ibadan is the capital of Oyo state, Nigeria and it is the third largest city in Nigeria.

### Design of the Study

This was a single arm non-randomized intervention study.

### Principles for Recruitment

#### Inclusion/Exclusion Criteria

The inclusion criteria for the study were: both male and female alcohol users of age ≥15 years and permanent residents of study areas. The exclusion criteria were non-users of alcohol of age <15 years, not willing to get alcohol intervention, and not a perma-nent resident of the study areas.

### Procedure

#### Sample Selection

A systematic stratified sampling method was used to select two semirural local governments in Ibadan between October 2010 and April 2011. The selected local governments were Lagelu local government (local government A) and Akinyele local government (local government B) areas of Ibadan. They were so classified according to the National Population Commission in Nigeria based on population and fund allocation.

In the first stage, all the 11 LGA were classified into urban or semirural each. There are five urban local governments and six semirural local government areas in Ibadan. In the second stage, two local governments were randomly chosen from the six semirural local government areas. In the third stage, four enumeration areas were systematically selected as clusters in each of the two local governments. The fourth stage involved the mapping and numbering of all buildings in each of the selected enumeration areas. All households within each building were serially listed in the form specifically designed for the purpose. After getting the list of the households, simple random sampling was used to identify the households that fell within the sample. Regular households were distinguished from institutional households. This approach was used to select a representative sample of all the members of the household. All eligible respondents, who were 15 years and above in each regular household, were selected and interviewed by CHEW using the questionnaires including ASSIST after they gave permission/consent. The CHEW also carried out the intervention as appropriate. After administration of the sociodemographic questionnaire, participants were also screened with ASSIST.

#### Prevalence of Current Alcohol Use

Prevalence of current alcohol use was obtained from the modification of Q2, which states, in the past 3 months, how often have you used alcohol? (Responses = “never,” “once or twice,” “monthly,” “weekly,” “daily/almost daily”). For the purpose of this study, cur-rent alcohol use was regarded as use in the preceding 30 days.

#### ASSIST Scoring

ASSIST scores were generated from questions 2–7 (Q2–Q7) on the ASSIST questionnaire, which elicited information on alcohol use in past 3 months. Each question on the ASSIST has a set of responses to choose from and each response has a numerical score. At the end of the interview, scores from Q2 to Q7 were added together to produce an ASSIST-risk score.

#### Intervention

The intervention was given at baseline and 3 months following baseline intervention. Assessments were carried out at baseline, 3 months, and 6 months. The participants received the level of risk-appropriate interventions using the ASSIST-linked brief intervention for hazardous and harmful substance use manual for use in primary care (26).

##### ASSIST Scoring

Low-risk clients (scores 0–10) were those at a lower risk of problems related to their alcohol use. While they may use alcohol occasionally, they were not at the time of the interview, experiencing any problems related to their alcohol use and were at a lower risk of developing problems related to their alcohol use in the future with their current pattern of use. This group was given brief advice.

Moderate-risk clients (scores 11 and 26) were at moderate risk of health and other problems and may be experiencing some of these problems now. Continuing use in this way indicated a likelihood of future health and other problems, including the possibility of dependence. The risk is increased for those with a past history of substance use related problems and dependence.

High-risk clients (scores 27 or higher) were those at high risk of dependence or is dependent on alcohol and is probably experiencing health, social, financial, legal, and relationship problems as a result of their alcohol.

#### Intervention

The ASSIST feedback report card was completed at the end of the ASSIST interview and was used to provide personalized feedback to the client about their alcohol use. The process of brief intervention was initiated by asking the client if he was interested in seeing how the scored questionnaire was completed and how the ASSIST-risk scores were computed in the boxes provided in the front of the ASSIST feedback report card. This was given to the client to have in his possession as a reminder of what has been discussed.

Lower risk clients received treatment as usual and were given feedback about their scores. Abstainers were also encouraged to remain the way they had been.

Moderate risk clients received a 3–15 min brief intervention which comprised of giving feedback to clients using the ASSIST feedback report card using simple motivational interviewing techniques. Clients were also given self-help strategies for cutting down or stopping alcohol use, substance use guide booklet, a copy of their ASSIST feedback report card, and specific drug information for keeping.

The brief intervention was also given to high-risk clients. This was to encourage clients to have a detailed clinical assessment and to negotiate a specialist treatment for their alcohol use.

In summary, the intervention had feedback and discussion on potential effects of alcohol consumption, emphasis on personal responsibility for the choice of behavioral change, a menu of options for a change strategy, the use of empathy, and encouragement of self-efficacy for change. For high-risk users, the interventionists were aware of the schedule and routine, and guidelines for treatment at the specialist addiction services at the University College Hospital, Ibadan, Nigeria.

#### Training of CHEW in ASSIST-Linked Intervention

The interventionists consisted of trained CHEW who conducted the baseline assessments, i.e., assessments at 3 and 6 months and also delivered the interventions to the recruited persons in the community. All CHEW who consented to follow the study protocol received formal training (duration) before data collection and supervision. The training took a practical, system approach and aimed to facilitate the implementation of SBIRT in the community and primary health care clinics. The training curriculum contained modules that raised and addressed practical issues that pertained to implementing the programme.

For identification of alcohol use problems in the community, and for brief intervention and referral for treatment, the WHO ASSIST-linked brief intervention and referral for treatment package for hazardous and harmful alcohol use were used (26).

To prevent against a drift by the CHEW interventionist, the brief intervention and referral for treatment were completely manualized, including the motivational interview protocol and used to guide the CHEW interventionist through the session content (16).

#### Intervention Quality Assurance

Assessment quality control was in charge of the first author. Fidelity was maintained by an audio tape recording of intervention sessions, the interventionist checklists and these were reviewed by the first author who provided feedback to the CHEWs. Process evaluation included a review of a random sample of 10% of the assessments and intervention audiotapes by the study coordinator for fidelity to protocol. The evaluation also involved the review of the study data to ensure fidelity of the intervention in the community setting.

### Outcome Measures

- *Baseline*: All eligible persons completed baseline measures that included a demographic questionnaire and the ASSIST (27). All consenting 15 years and older persons had administered a sociodemographic questionnaire to elicit sociodemographic characteristics from the respondents such as age, marital status, socioeconomic class, and years of education. The ASSIST was developed for screening of alcohol and drug use, especially in high-prevalence settings (27). For the purpose of this study, current alcohol use was regarded as use in preceding 30 days. The primary outcome measure used in this study was the change in the ASSIST alcohol use scores from baseline to follow-up.
- *Follow-up*: The same measures used above were administered at follow-up to the participants at 3 months and again at 6 months. To prevent loss to follow-up and retain participant in the program, bulk SMS messages were sent 2 weeks prior to interviewing date. These messages reminded them that they were in the programme.

### Data Analysis

For our univariate analysis, the association between sociodemographic variables and current alcohol use was determined using the Pearson’s chi square statistics. Differences in prevalence of alcohol use across study point were compared using Friedman test and *post hoc* pairwise comparison carried out using the Wilcoxon signed-rank test. The repeated measures ANOVA was used to assess the presence of any significant difference in the mean ASSIST scores at baseline, 3 months, and 6 months. *Post hoc* pairwise comparisons were carried out using paired *t*-test.

Linear regression analyses were carried out using variables that were significant during univariate analysis to determine association with alcohol use. All analyses were by SPSS version 13.0 (28).

## Results

A total of 1,329 youths and adults were selected and invited to participate in this study. Out of these, about 1,213 completed the questionnaires, giving a response rate of 91.3%. At baseline, responses were incomplete in 10 questionnaires and so final analysis was carried out on 1,203 questionnaires at baseline. At 3 months, an analysis was carried out on 1,199 respondents and on 1,195 at 6 months.

The mean age of respondents at baseline was 24.45 ± 9.23 years, 51.8% were males, 66.2% were married, 47.4% had at least some secondary education, and 49.7% were of a low-average socio-economic group. Current alcohol use increased with increasing age, *p* = 0.02, was more common in males, *p* = 0.003, among the unmarried, *p* < 0.01, among those with formal education, *p* = 0.003, and in those from low socioeconomic group, *p* = 0.01 (Table 1).

**Table 1:** **Sociodemographic and other characteristics of respondents at baseline *N* = 1,203**.

Variables	Total *N* = 1,203	%	Current alcohol use (*N* = 285)	%	χ^2^	Sig
**Age**						
<25	508		133	26.2	13.5	0.02
25–34	256		61	23.8		
35–44	158		36	22.8		
45–54	120		21	17.5		
55–64	111		15	13.5		
>64	50		6	12.0		
**Gender**						
Male	623		170	27.3	8.8	0.003
Female	580		115	19.8		
**Marital status**						
Married	796		170	21.4	6.7	<0.01
Not married	407		115	28.3		
**Setting**						
Local government A	487		78	16.0	0.2	0.7
Local government B	716		107	14.9		
**Education**						
0	119		44	37.0	13.0	0.003
1–6	431		101	23.4		
7–12	570		120	21.1		
>12	83		20	24.1		
**Socioeconomic group**						
Low	513		146	28.5	11.4	0.01
Low average	598		122	20.4		
High average	63		12	19.4		
High	29		5	17.2		

*Prevalence of current alcohol use was obtained from the modification of Q2, which states, in the past 3 months, how often have you used alcohol? (Responses = “never,” “once or twice,” “monthly,” “weekly,” “daily/almost daily”). For the purpose of this study, current alcohol use was regarded as use in preceding 30 days*.

At baseline, the prevalence of current alcohol use was 23.7%. At 3 months, the prevalence of current alcohol use was 17.1%. While at 6 months, the prevalence of current alcohol use was 13.6%. There was a statistically significant difference in the prevalence of alcohol use postintervention, compared with prevalence at baseline χ^2^(2) = 4.2, *p* = 0.01. *Post hoc* analysis with Wilcoxon signed-rank tests was conducted with a Bonferroni correction applied. Thus, there was a significant reduction between the baseline prevalence and 3 months prevalence (*Z* = −9.61, *p* < 0.001) and also a significant reduction in 6 months prevalence compared with 3 months prevalence (*Z* = −4.5, *p* = 0.001; Table 2).

**Table 2:** **Prevalence of alcohol use at baseline, 3 months, and 6 months**.

Alcohol use	Baseline		3 months		6 months		χ^2^	p

	*N*	%	*N*	%	*N*	%		
Yes	285	23.7	205	17.1	163	13.6	df(2) 4.2	0.01
No	918	76.3	994	82.9	1,032	86.4		

With respect to those who had low-risk scores, a repeated measures ANOVA with a Greenhouse–Geisser correction showed that the mean ASSIST score significantly reduced between time points [*F*(1.116, 2.595) = 366.692, *p* < 0.001]. *Post hoc* tests using the Bonferroni correction revealed that this difference was due to a significant reduction in the mean ASSIST scores at 3 months vs. baseline, *p* < 0.001, and a further reduction in the mean ASSIST scores at 6 months compared with that at 3 months *p* < 0.001 (Table 3).

**Table 3:** **ASSIST score at baseline, 3 months, and 6 months**.

Health risk	Baseline *N* = 1,203		3 months *N* = 1,199		6 months *N* = 1,195		Baseline vs. 3 months	3 months vs. 6 months
Score	*N*	Mean (SD)	*N*	Mean (SD)	*N*	Mean (SD)	Sig	Sig
0–10	88	8.41 (1.58)	116	5.05 (2.05)	113	2.21 (1.01)	<0.001	<0.001
11–26	161	20.52 (5.42)	69	15.51 (4.46)	35	13.93 (3.66)	<0.001	0.06
27+	38	38.38 (6.06)	20	26.83 (5.71)	15	24.74 (7.48)	<0.001	0.4
All respondents	285	27.21 (7.21)	205	22.87 (4.06)	163	22.03 (4.99)	0.001	0.09

For those in the moderate risk category, a repeated measures ANOVA with a Greenhouse–Geisser correction showed that mean ASSIST score significantly reduced between time points [*F*(1.466, 24.841) = 40.177, *p* < 0.001]. *Post hoc* tests using the Bonferroni correction revealed that this difference was due to a significant reduction in the mean ASSIST scores at 3 months vs. baseline, *p* < 0.001, however, there was no significant difference in the mean scores at 6 months compared with that at 3 months, *p* = 0.06 (Table 3).

With regard to participants with severe risk of harm, a repeated measures ANOVA with a Greenhouse–Geisser correction showed that mean ASSIST score significantly reduced between time points [*F*(1.429, 39.451) = 36.242, *p* < 0.001]. *Post hoc* tests using the Bonferroni correction revealed that this difference was due to a significant reduction in the mean ASSIST scores at 3 months vs. baseline, *p* < 0.001, but between the third and sixth month, the difference was not significant, *p* = 0.4 (Table 3).

Among all respondents, a repeated measures ANOVA with a Greenhouse–Geisser correction showed that mean ASSIST score significantly reduced between time points [*F*(1.541, 34.092) = 53.241, *p* < 0.001]. *Post hoc* tests using the Bonferroni correction revealed that this difference was due to a significant reduction in the mean ASSIST scores at 3 months vs. baseline, *p* = 0.001, but not at 3 vs. 6 months, *p* = 0.09 (Table 3).

Linear regression analysis revealed that at baseline, older age group, female gender, a high-average socioeconomic group, high socioeconomic group were protective factors, while, being unmarried was a risk factor.

At 3 months, older age group, female gender, a high-average socioeconomic group, a high socioeconomic group were protective factors, while and being unmarried was a risk factor.

At 6 months, older age group, female gender, high-average socioeconomic group, high socioeconomic group were protective factors, while, being unmarried was a risk factor (Table 4).

**Table 4:** **Odd ratio for current alcohol use**.

	Baseline			3 months			6 months		
Variation	OR	CI	Sig	OR	CI	Sig	OR	CI	Sig
**Age**									
<25	1			1		1			
25–34	0.58	0.33–1.00	0.05	0.57	0.34–1.00	0.05	0.53	0.29–0.99	<0.05
35–44	0.59	0.22–0.94	<0.05	0.50	0.23–0.99	<0.05	0.49	0.28–0.93	0.03
45–54	0.41	0.11–0.88	0.04	0.43	0.29–0.77	0.03	0.24	0.07–0.71	0.01
55–64	0.35	0.09–0.87	0.04	0.32	0.09–0.67	0.01	0.12	0.03–0.49	0.001
>64	0.21	0.08–0.75	0.02	0.12	0.02–0.32	0.001	0.09	0.01–0.34	<0.001
**Gender**									
Male	1			1			1		
Female	0.32	0.008–0.62	0.01	0.22	0.009–0.55	<0.01	0.12	0.03–0.39	<0.01
**Marital status**									
Married	1			1			1		
Not married	3.32	1.66–6.12	0.01	3.07	1.21–5.02.	<0.01	1.95	1.09–4.22	0.01
**Socioeconomic group**									
Low	1			1			1		
Low average	0.89	0.29–1.44	0.16	0.49	0.09–1.04	0.06	0.46	0.04–1.06	0.07
High average	0.32	0.09–0.87	0.03	0.01	0.01–0.47	0.02	0.06	0.01–0.33	0.01
High	0.24	0.08–0.72	0.02	0.11	0.01–0.49	0.01	0.03	0.004–0.11	0.001

## Discussion

To our knowledge, this is the first study to evaluate the effectiveness of ASSIST-linked SBIRT on harmful and hazardous alcohol use among youths and adults in Nigerian semirural settings and probably in sub-Saharan Africa. The study was conducted in two representative different provinces in Ibadan, the largest city in West Africa. These diverse settings underlie a strength in the generalizability of the findings.

A major finding in the present assessment was that the rate of alcohol use reduced significantly between baseline and 6 months, so also was the mean ASSIST scores. Even though McCambridge and Kypri (29) reviewed that later the self-report behavior appeared to be altered by simply answering questions on drinking in SBI trials, our finding overall, suggests that the provision of a brief intervention and referral can help reduce levels of hazardous and harmful alcohol use in youth as well as adult dwellers in semirural settings in Nigeria. We, therefore, argue that the risks of documented adverse consequences of problematic alcohol use such as alcohol dependence, cancers, and injuries could be mitigated with this intervention. This could be particularly advantageous in semirural or rural settings, where there is limited access to health care, and the probability of identification of such adverse consequences is low.

Although there was a significant reduction in the prevalence of alcohol use as well as the mean ASSIST scores between the baseline and 6 months among all respondents, *post hoc* analyses showed that the reductions were for baseline assessment vs. assessment at 3 months and not for the assessments at 3 vs. 6 months. By implication, a considerable proportion of youths and adults in this study might not be in the preparatory or action stage of change. Unfortunately, we did not match the intervention to different stages of change. This is important because readiness to quit is a determinant of the continued alcohol consumption. Our findings underscore the significance of matching treatment to the patient’s stage of change. This is paramount to achieve an efficacy of the intervention (30). Another possible explanation, using the Bronfenbrenner ecological model could be that factors such as enabling or disabling individual, familial, and community-level conditions, were not measured and taken into account in our study. There is a need for future studies to examine the impact of different stages of change and ecological variables on the effectiveness of SBIRT on alcohol use in semirural settings.

Studies of brief intervention delivery by CHEW in rural or semirural community settings have not been reported in a significant proportion of developing countries including sub-Saharan Africa. However, we found that this task-shifting approach (31), is applicable in alcohol cessation programs similar to the way it has been found effective in stepped care approach to manage depression in primary care setting in Nigeria (24), and outside the field of mental health (32). These CHEWSs were more readily available, have fewer qualifications, and were trainable. Screening, brief intervention, and referral to treatment delivery by CHEW seems to be feasible and implementable with fidelity in the Nigerian semirural community settings. It will be interesting to see whether future studies will confirm this in similar settings in Nigeria and other developing countries.

This current study is important in three ways: (1) it focused on alcohol; (2) SBIRT was deliverable by CHEW rather than by clinicians and adds to the literature on the effectiveness of task shifting in community mental health; and (3) it enrolled people in the community with poor access to orthodox medicine and who might not seek treatment rather than those who went to the hospital or were admitted in emergency settings.

Our study was limited by a number of factors. One, we could not explore the impact of stages of change on unhealthy alcohol use in a rural community setting. We, therefore, suggest that future studies address the potential influence of stages of change on alcohol use reduction. Two, we did not examine alcohol cessation in relation to SBIRT. Three, there was no control group. Four, the data were all self reports with no toxicological assessments. Five, we also did not allocate any diagnosis to the alcohol users; therefore, it was difficult to determine the effect of the intervention on any specific diagnostic category of alcohol user. Six, the attrition observed across the study should be recognized as an important limitation. Also, the non-randomized nature of the study makes it susceptible to selection, performance, and attrition bias. However, we attempted to address these issues by reporting all the essential information on the methodology and results in accordance with CONSORT guidelines. The absence of a control group for the assessments and interventions delivered these CHEWS, is another limitation.

Given these limitations, we conclude herein that the delivery of ASSIST-linked SBIRT for unhealthy alcohol is feasible by CHEW in semirural settings. This preventive health model could be integrated into substance abuse services for Nigerian rural and semirural communities.

## Ethics Statement

Ethical approval for the study was received from the Ethics and Research Committee of the Ministry of Health, Oyo State, Nigeria. Assent was obtained for participants between 15 and 18 years and consent from those over 18 years of age.

## Author Contributions

VL was involved in study design, data collection, analysis of data, and manuscript writing. BAO was involved in data collection, and final manuscript writing and editing. OOA was involved in data collection, manuscript writing, and editing.

## Conflict of Interest Statement

The authors declare that the research was conducted in the absence of any commercial or financial relationships that could be construed as a potential conflict of interest. The reviewer, GT, and handling Editor declared their shared affiliation, and the handling Editor states that the process nevertheless met the standards of a fair and objective review.
